# Ferroptosis in diabetic cardiomyopathy: from its mechanisms to therapeutic strategies

**DOI:** 10.3389/fendo.2024.1421838

**Published:** 2024-11-11

**Authors:** Meimei Tian, Xinli Huang, Min Li, Pingping Lou, Huijie Ma, Xinli Jiang, Yaru Zhou, Yan Liu

**Affiliations:** ^1^ Department of Endocrinology, The Third Hospital of Hebei Medical University, Shijiazhuang, Hebei, China; ^2^ Department of Pathophysiology, Hebei Medical University, Shijiazhuang, Hebei, China; ^3^ Department of Physiology, Hebei Medical University, Shijiazhuang, Hebei, China; ^4^ Hebei Collaborative Innovation Center for Cardio-Cerebrovascular Disease, Shijiazhuang, China; ^5^ Department of Ophthalmology, The Third Hospital of Hebei Medical University, Shijiazhuang, Hebei, China

**Keywords:** diabetic cardiomyopathy, iron metabolism, ferroptosis, mitochondria, therapy

## Abstract

Diabetic cardiomyopathy (DCM) is defined as structural and functional cardiac abnormalities in diabetes, and cardiomyocyte death is the terminal event of DCM. Ferroptosis is iron-dependent oxidative cell death. Evidence has indicated that iron overload and ferroptosis play important roles in the pathogenesis of DCM. Mitochondria, an important organelle in iron homeostasis and ROS production, play a crucial role in cardiomyocyte ferroptosis in diabetes. Studies have shown some anti-diabetic medicines, plant extracts, and ferroptosis inhibitors might improve DCM by alleviating ferroptosis. In this review, we systematically reviewed the evidence of ferroptosis in DCM. Anti-ferroptosis might be a promising therapeutic strategy for the treatment of DCM.

## 1 Introduction

The global prevalence of diabetes mellitus (DM) is increasing. Data from the International Diabetes Federation (IDF) has indicated that the global prevalence of DM was estimated to be 10.5% (536.6 million) of adults in 2021 (1). Cardiovascular disease is the leading cause of death in diabetic patients (2). Early in 1972, Rubler et al. described a pathological cardiac alteration in DM patients, which was characterized by ventricular hypertrophy and fibrosis, and termed diabetic cardiomyopathy (DCM) (3). Currently, DCM is defined as structural and functional cardiac abnormalities in diabetes, which cannot be explained by hypertension, coronary artery heart disease, valvular heart disease, or other heart diseases. DCM is the leading cause of heart failure and death in DM patients (4).

The death of cardiomyocytes is the terminal event of DCM (4–6). Ferroptosis, which was first described in 2012 by Dixon et al., is iron- and lipotoxicity-dependent cell death, and controlled by multiple pathways involved in iron accumulation, lipid peroxidation, or a disturbed antioxidant system (7). Evidence from recent years has indicated that ferroptosis participates in many heart diseases, including myocardial infarction (8), cardiac ischemia/reperfusion (I/R) injury (9), heart failure (10), myocardial hypertrophy (11), sepsis (12), and doxorubicin-induced heart injury (13). Therefore, therapies targeting ferroptosis or iron overload might be promising for cardiac diseases (14–17).

Iron overload and ferroptosis have been found to be closely correlated with diabetes and its complications (18, 19). Tissue iron overload causes increased ROS through the Fenton response, exacerbating diabetic cardiovascular complications (20). Therefore, in the present review, we summarized the related data about iron metabolism and ferroptosis and discussed their role in the pathogenesis of DCM, which may provide new evidence for the pathogenesis of DCM and its targeted therapy.

## 2 Iron and DCM

**Figure 1:**
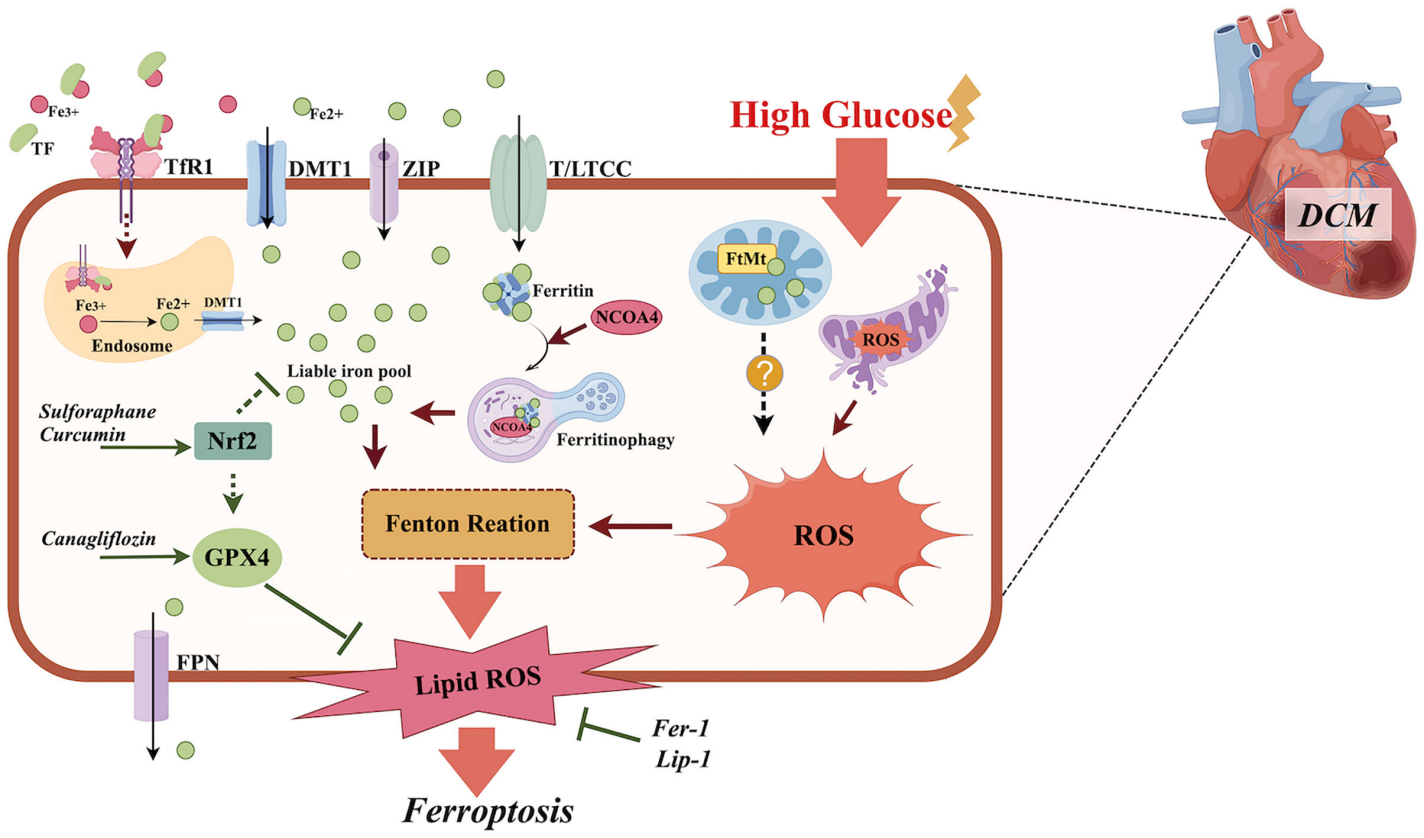
The role of ferroptosis in the pathogenesis of DCM. Diabetes or high glucose may cause iron overload, mitochondria disorder, increased NCOA4-mediated ferritinophagy, and an impaired anti-oxidative Nrf2/GPX4 pathway in cardiomyocytes, which all contribute to increased lipid peroxidation and ferroptosis. Ferroptosis inhibitors Fer-1 and Lip-1, anti-diabetic medicine canagliflozin, and the plant extract sulforaphane all exhibit anti-ferroptotic effects in DCM by suppressing lipid peroxidation, activating Nrf2 and promoting Xc-/GSH/GPX4 axis separately. TF, transferrin; TfR1, transferrin 1 receptor; DMT1, divalent metal transporter 1; ZIP, Zrt-, Irt-like Proteins; Nrf2, nuclear factor erythroid 2-related factor 2; NCOA4, nuclear receptor coactivator 4; GSH, glutathione; GPX4, glutathione peroxidase 4; L/TTCC, L/T-type calcium channel; FtMt, mitochondrial ferritin; ROS, reactive oxygen species; Fer-1, ferrostatin–1; Lip-1, liproxstatin–1. The figure was created by Figdraw.

Iron is essential for many physiological processes including oxygen transport and mitochondrial energy metabolism. The iron enters the cardiomyocytes by chelating to transferrin, subsequently binding to the transferrin 1 receptor (TfR1) (21), but also through other routes including the T-type calcium channel (TTCC), divalent metal transporter 1(DMT1) (22), the L-type calcium channel (LTCC) (23), and Zrt-, Irt-like proteins (ZIP) 8 and 14 (24). Intracellular iron is utilized, stored bound to cytoplasmic ferritin, or imported by mitochondria. Excess iron can be extruded from cardiomyocytes by ferroportin (FPN) (**Figure 1**).

Iron overload is an excessive accumulation of iron, which has been found to be associated with insulin resistance, diabetes, and its complications (20, 25). In human studies, iron overload, which is demonstrated by serum iron levels, was associated with increased fasting plasma glucose and the occurrence of type 2 diabetes mellitus (T2DM) (26), and is positively associated with higher visceral fat mass in T2DM patients (27). Iron homeostasis is important to maintain cardiac function. Cardiac iron overload has been found to participate in the pathogenesis of 5-fluorouracil(5-FU) induced cardiotoxicity (28), doxorubicin-induced cardiomyopathy (29), and cardiac I/R injury (30). It has been suggested that iron overload may induce insulin resistance in cardiomyocytes (31), manipulate cardiac calcium regulation (32, 33), cause reactive oxygen species (ROS) accumulation and lipid peroxidation (34, 35), and therefore lead to cardiac dysfunction (36). Although insulin resistance plays a key role in the pathogenesis of DCM, whether an impaired insulin signaling pathway could cause iron overload in cardiomyocytes has almost never been reported in the literature.

In 2016, Li et al. examined the myocardial iron content using the aromatic absorption spectrophotometry method in diabetic rats induced by high fat diet and streptozotocin (STZ) injection, and no alteration was found between the diabetic rats and control rats (37). In another study performed in type 2 diabetic mice by Wang et al., labile iron content was significantly increased in myocardial tissue (38). Furthermore, in another study in STZ-induced diabetic rats, Fe2+ content was found to be increased in the heart tissue, indicating iron overload existed in DCM (39). An *in vitro* study conducted by Li et al. (40), found increased Fe2+ content in H9C2 cells treated with high glucose. Therefore, iron overload was found in DCM and might be correlated with the pathogenesis of DCM (**Table 1**).

**Table 1:** Iron content alterations in diabetic models.

Animals/cells	Iron measurement/method	Trend	Reference
High fat diet/STZ-induced diabetic rats	Total myocardial iron, atomic absorption spectrophotometry	no change	37
Type 2 diabetic mice	Labile iron levels/ Iron Colorimetric Assay Kit	increase	38
STZ-induced diabetic rats	Intracellular ferrous ion (Fe2+)/ Iron assay kit	increase	39
H9c2 cells	Intracellular ferrous ion (Fe2+)/Iron assay kit	increase	40

## 3 Ferroptosis and DCM

Iron overload may trigger ferroptosis, a unique form of non-apoptotic cell death, which is characterized by iron-dependent lipid peroxidation (41). Cells that undergo ferroptosis exhibit malformed mitochondria, a decreased crest, membrane concentration, rupture of the outer membrane, and an absence of features of apoptosis. The main cause of ferroptosis is the depletion of glutathione and impaired function of phospholipid peroxidase glutathione peroxidase 4 (GPX4) which can protect cell membranes from peroxidative damage (7, 42). Ferroptosis participates in many cardiomyopathies including doxorubicin-induced cardiomyopathy (29), myocardial infarction (43), and heart failure (44). In diabetes, increased advanced glycation end-products (38), lipid peroxidation, and oxidative stress (45) all participate in the pathogenesis of DCM, which are also the triggers for cellular iron overload and ferroptosis. The expression of ferroptosis-promoting genes was increased in the heart tissue from STZ-induced diabetic mice (46) and ferroptosis was found in T2DM mice with DCM (38). A ferroptosis-promoting gene profile was also found by Gawargi et al. (47) in the heart tissue of diabetic patients with heart failure. Furthermore, ferroptosis inhibition led to improved cardiac contract function in T2DM mice (38). Therefore, ferroptosis participates in DCM and could be an intervention target in DCM therapy.

Ferroptosis is a complicated process in which many regulators and pathways are involved (48, 49). The detailed mechanisms underlying ferroptosis in DCM have been investigated but are quite limited. Nuclear factor erythroid 2-related factor 2 (Nrf2) is an important transcriptional factor and participates in multiple biological processes including anti-oxidation (50). It has been found that the activation of Nrf2 may improve DCM (51–53) and this effect might be partly via anti-ferroptotic effects. Wang et al. found that sulforaphane can increase ferritin expression in myocardial tissue by activating Nrf2, which may inhibit advanced glycation end-product-induced ferroptosis in DCM (38). Nrf2/GPX4, Nrf2/HO-1 pathway activation could inhibit ferroptosis and thus alleviate high glucose-induced cardiomyocyte injury (54–56) (**Figure 1**). Recently, novel evidence has indicated that alterations in the intestinal microbiota (57) reduced cardiac expression of retinol dehydrogenase 10 (58) and upregulated lysine acetyltransferase 2 A (Kat2a) (59), both of which participate in ferroptosis in DCM and provide more therapy targets for DCM.

Mitochondria are not only the organelle that produces ROS but are also pivotal for iron metabolism and ferroptosis (60). Cardiomyocytes need a continuous energy supply to maintain their beating. Mitochondria, as the energy factories, are crucial for maintaining heart function. It has been suggested that mitochondrial dysfunction participates in the occurrence and the devolvement of DCM (61, 62). The role of mitochondria in iron overload and ferroptosis has been studied but is still complicated. Iron overload caused cardiac and mitochondria dysfunction in rats (63) and led to mitochondrial iron accumulation, an increase in mitochondrial ROS, and ferroptosis in cardiomyocytes (64). Data from an *in vitro* study indicated that oxidative stress, which was induced by a tert-butyl hydroperoxide treatment, induced mitochondrial iron overload and cardiomyocyte ferroptosis by targeting the Bach1-HO-1 pathway (65). Furthermore, in doxorubicin-induced cardiomyopathy, doxorubicin triggered iron accumulation in mitochondria, which further caused cardiomyocyte ferroptosis (29, 66). Therefore, mitochondrial iron overload is crucial for cardiomyocyte ferroptosis; however, detailed evidence of its role in DCM has never been reported.

The iron-sulfur cluster (ISC) is an ancient and conserved cofactor that is mainly assembled in mitochondria, and the loss of its synthesis leads to iron overload and ferroptosis. Frataxin is a mitochondrial ISC-related protein and an important regulator for ferroptosis (67). Patients with reduced frataxin expression have an increased risk of diabetes mellitus (68) and cardiomyopathy (69). Furthermore, decreased frataxin expression has been found to cause cell ferroptosis in adipose tissue (70) and heart tissue (71).

Mitochondrial ferritin (FtMt) is structurally similar to ferritin-heavy chains but has lower ferroxidase activity. FtMt overexpression can lead to intracellular iron redistribution by transferring iron from the cytoplasm into mitochondria, consequently leading to reduced iron content in the cytoplasm (72, 73). It has been found that FtMt could protect cells from oxidative stress by regulating the mitochondrial labile iron pool and ROS production (73). Mice with a FtMt deficiency are more sensitive to cardiomyocyte damage caused by doxorubicin (74) and fatigue (75), indicating that cardiomyocytes with FtMt deficiency are more prone to injury. FtMt overexpression could inhibit oxidative stress-induced ferroptosis through the inhibition of mitochondrial iron overload and ROS in cardiomyocytes (65). Wang et al. found that the overexpression of FtMt could ameliorate oxidative stress and ferroptosis in osteoblasts caused by high glucose (76). Unfortunately, thus far, there is no data available on the role of FtMt in DCM.

Mitophagy refers to the targeted phagocytosis and destruction of mitochondria by the cellular autophagy apparatus and is considered to be the main mechanism of mitochondrial quality control. Studies on DCM have suggested that decreased mitophagy may lead to the accumulation of abnormal mitochondria, and result in increased intracellular oxidative stress, which triggers the occurrence and development of DCM (77). Improving mitophagy can improve the risk of developing DCM (78). Studies conducted in non-cardiomyocytes have found that activating mitophagy may inhibit ferroptosis. Li et al. found that activating PINK1-Parkin-dependent mitophagy could protect cells from CISD3-induced ferroptosis (79). Therefore, whether reduced mitophagy may trigger ferroptosis in DCM is a promising research topic that needs to be studied.

## 4 Ferritinophagy and DCM

Ferritin is a cytosolic storage protein complex consisting of ferritin heavy-chain (FTH1) and light-chain (FTL) subunits, responsible for intracellular iron storage (80), and exerts antioxidant effects by isolating redox-active iron. Ferritin can affect cell susceptibility to ferroptosis (81). Ferritinophagy is a selective form of ferritin autophagy degradation whose overactivation induces increased degradation of ferritin which binds to iron, and increased iron release leads to iron overload, leading to cellular ferroptosis. Thus, ferritinophagy plays an important role in the regulation of ferroptosis by regulating intracellular iron balance (82).

Nuclear receptor coactivator 4 (NCOA4) is a selective ferritinophagy cargo receptor that directly recognizes and binds to FTH1 and transports ferritin to autophagosomes for lysosomal degradation and iron release (83). In non-cardiomyocytes, NCOA4 knockdown (84) or inhibition of the NCOA4-FTH1 association (85) was found to inhibit ferroptosis. Cardiac NCOA4 expression was significantly increased while GPX4 expression was decreased in diabetic rats (39) and activated NCOA4-mediated ferroptinophagy and ferroptosis were found in the heart tissue of db/db mice (86). NCOA4 knockdown or inhibition alleviated ferroptosis in a DCM model *in vitro (* 87) and *in vivo (* 86), suggesting that increased ferritinophagy plays an important role in the occurrence of DCM (**Figure 1**).

## 5 Hypoglycemic drugs, ferroptosis, and DCM

### 5.1 Metformin

As a classic hypoglycemic drug, metformin has been previously found to be protective in DCM by alleviating apoptosis (88), improving autophagy, inhibiting pyroptosis (89), and alleviating fibrosis (90). In doxorubicin-induced cardiotoxicity mouse models, metformin treatment inhibited ferroptosis and improved cardiac function by activating AMP-activated protein kinase (AMPK)α2 phosphorylation (13). In the study by Wu et al., metformin alleviated cardiac I/R damage *in vivo* and *in vitro* by relieving non-heme iron content and ferroptosis by activating AMPKα and inhibiting nicotinamide adenine dinucleotide phosphate oxidase 4 expression (91). However, thus far, there is no data available on whether metformin may alleviate ferroptosis in DCM.

### 5.2 Glucagon-like peptide-1 receptor agonists

Glucagon-like peptide-1 receptor agonists (GLP–1RAs) have attracted much attention in recent years for their cardiac protective effects. Studies have found that liraglutide can improve cardiac function in diabetic patients (92) and improve the endoplasmic reticulum stress of cardiomyocytes in diabetic animals (93). In studies of db/db diabetic mice, liraglutide has been found to reduce iron overload in the liver as well as the hippocampus, and reduce ferroptosis (94, 95). In a nationwide register-based study performed by Bain et al., GLP-1RA administration was found to be associated with lower circulating ferritin levels in patients with type 2 diabetes and hemochromatosis (96). Therefore, while alleviating ferroptosis might partly contribute to the cardio-protective effects of GLP-1RAs in DCM, more investigations are warranted.

### 5.3 Sodium-glucose co-transporter-2 inhibitors

The cardiovascular benefits of sodium-glucose co-transporter-2 (SGLT2) inhibitors have been increasingly documented in recent years. Evidence from *in vivo* and *in vitro* studies has indicated that SGLT2 inhibitors, such as empagliflozin and dapagliflozin, can improve DCM by attenuating oxidative stress (97, 98). Empagliflozin exhibited anti-ferroptotic effects in high glucose-treated muscle C2C12 cells by restoring the expression of GPX4 (99) and in diabetic kidney disease models by activating Nrf2 (100). Thus far, only canagliflozin has been found to inhibit ferroptosis in DCM by balancing cardiac iron homeostasis, promoting Xc-/glutathione(GSH)/GPX4 axis (101), and activating the AMPK pathway (102) (**Figure 1**).

### 5.4 Dipeptidyl peptidase 4 enzyme inhibitors

The protective effects of dipeptidyl peptidase 4 (DPP-4) inhibitors on the DCM have been emerging in research in recent years. For example, linagliptin improved cardiac function in diabetic mice by inhibiting the NF-κB signaling pathway and relieving the cardiac inflammatory response by targeting the NOD-, lrr-, and pyrin domain-containing protein 3/apoptosis-associated speck-like protein containing a caspase recruitment domain (Nlrp3/ASC) inflammasome (103, 104). Sitagliptin was found to attenuate DCM by attenuating myocardial apoptosis, inflammation, and nitroxidative stress by targeting the liver kinase B1/AMPK/Protein kinase B (LKB-1/AMPK/Akt) and Janus kinase/signal transducers and activators of transcription (JAK/STAT) pathways and promoting cardiomyocyte autophagy separately (105–107). Furthermore, alogliptin could improve mitochondrial function in DCM (108). However, the effect of DPP-4 inhibitors on iron metabolism or ferroptosis has been scarcely investigated. In brain tissue, vildagliptin has been found to reduce iron deposition and inhibit ferroptosis following intracerebral hemorrhage (109). The role of DPP-4 inhibitors on ferroptosis in DCM is an interesting subject that needs to be further explored.

### 5.5 Thiazolidinediones

Thiazolidinediones (TZDs), a class of peroxisome proliferator-activated receptor gamma (PPARγ) agonist, is the inhibitor of the ferroptosis marker ACSL4 (110), and has been found to prevent ferroptosis in many tissues and models including acute kidney injury (111), a ferroptosis mouse model (112), lung I/R injury (113), and renal fibrosis (114). However, in a diabetic rat model, TZD treatment was found to be detrimental as it caused cardiomyocyte ferroptosis and structural heart disorders (115).

Thus, in addition to their hypoglycemic effects, the effects of these drugs on ferroptosis in DCM need to be further explored (**Table 2**).

**Table 2:** The effects of hypoglycemic drugs on ferroptosis and DCM.

Drugs	DCM protective effects	Anti-ferroptotic effects in other tissues /models	Direct evidence of ferroptosis in DCM
Met	Yes (88–90).	DOX-induced cardiomyopathy (13); Cardiac I/R (91)	Not available
GLP-1RA	Yes (92, 93).	Diabetic liver and hippocampus (94, 95); Blood sample of T2DM patients (96)	Not available
SGLT2is	Yes (97, 98)	High glucose-treated muscle C2C12 cells (99); Diabetic kidney disease models (100)	Balanced cardiac iron homeostasis, promoted Xc-/ GSH/GPX4 axis (101); activated AMPK pathway (102)
DPP4-is	Yes (103–108).	Brain tissue following intracerebral hemorrhage (109)	Not available
TZDs	None	Acute kidney injury (111); Ferroptosis mice model (112); I/R induced lung injury (113); Renal fibrosis (114)	Caused cardiomyocyte ferroptosis and histoarchitectural disrrangements (115).

Met, Metformin; DOX, doxorubicin; I/R, ischemia/reperfusion; GLP-1RA, glucagon-like peptide-1 receptor agonist; SGLT2i, sodium glucose co-transporter-2 inhibitors; DPP4-is, dipeptidyl peptidase 4 enzyme inhibitors; TZDs, thiazolidinediones; Xc-/GSH/GPX4, system Xc−/glutathione peroxidase 4 /glutathione.

## 6 Plant extracts, DCM, and ferroptosis

### 6.1 Resveratrol

Resveratrol is a non-flavonoid polyphenol mainly found in a variety of fruits and vegetables, including peanuts, grapes, and berries. In recent years, much attention has been paid to the effects of resveratrol due to its antidiabetic and cardiovascular protective properties. Data have indicated that resveratrol, including its natural precursor polydatin, could alleviate DCM by improving mitochondrial function, alleviating oxidative stress, and inhibiting nuclear factor kappa B (NF-κB) activity (116, 117). Resveratrol has been found to inhibit cardiomyocyte ferroptosis in I/R models *in vivo* and *in vitro* by decreasing TfR1 while increasing GPX4 and FTH1 expressions, regulating of ubiquity specific peptidase 19 (USP19)-Beclin1 autophagy (118), and targeting the voltage-dependent anion channel 1/glutathione peroxidase 4 (VDAC1/GPX4) pathways (119). Whether its anti-ferroptotic effects exist in DCM needs to be further investigated.

### 6.2 Flavonoids

Flavonoids are natural plant polyphenolic phytochemicals and are widely found in fruits, nuts, vegetables, flowers, vegetables, and herbs. There is a large amount of evidence from *in vitro* and *in vivo* studies that indicates that flavonoids possess iron-chelating and antioxidant abilities (120). Flavonoids could improve DCM mainly through their anti-inflammatory and anti-oxidation effects (121). In recent years, much attention has been paid to the anti-ferroptotic effects of flavonoids (122–124) and studies have indicated that flavonoids could protect against ferroptosis-mediated tissue damage. Therefore, there are strong possibilities that flavonoids could achieve their DCM protective effects by alleviating ferroptosis. However, the existing research mainly focuses on liver and kidney injury, and the evidence for DCM is still lacking.

### 6.3 Sulforaphane

Sulforaphane is found in cruciferous vegetables and is a natural isothiocyanate compound. An activator of Nrf2, the literature has revealed the effects of sulforaphane on the amelioration of diabetic complications (125, 126) and cardiovascular disease (127). Studies performed in DCM models have shown that sulforaphane could improve cardiac function, cardiac hypertrophy, fibrosis, inflammation, and oxidative damage (38, 128–131). The anti-ferroptotic effects of sulforaphane have been found in diabetic livers (132), cardiac arrest and resuscitation (133), and myocardial I/R models (127). In DCM models, sulforaphane could inhibit cardiomyocyte ferroptosis by upregulating ferritin and SLC7A11 levels via AMPK-mediated Nrf2 activation (38), but more evidence on the effect of sulforaphane on DCM is still needed.

### 6.4 Curcumin

Curcumin is a polyphenolic compound extracted from the rhizomes of the turmeric plant and exhibits DCM protective effects through its antioxidant (134, 135) and anti-inflammatory (136) properties. Evidence has indicated its favorable effects on osteoarthritis (137), acute kidney injury (138), and cigarette smoke-caused lung epithelial injury (139) by alleviating ferroptosis. Zhang et al. (55) found that curcumin inhibited ferroptosis in cardiomyocytes by promoting the function of Nrf2 and increasing the expression of GPX4 and heme oxygenase-1 in DCM models.

### 6.5 Berberine

Berberine, an isoquinoline alkaloid isolated from the Chinese herb *Coptis chinensis* and other Berberis plants, has been found to alleviate DCM by preventing cardiac dysfunction and remodeling (140), being anti-fibrotic (141), interfering with lipidomic profiles (142), and inhibiting pyroptosis (143, 144). However, although a large amount of data has found that berberine could alleviate ferroptosis in many cells and models including islet beta cell loss in T1DM (145), a polycystic ovarian syndrome (PCOS) cell model (146), contrast-induced nephropathy (147), and bone loss induced by nonalcoholic fatty liver disease (148), no data are available on whether these anti-ferroptotic effects also participate in its DCM protective effects.

Therefore, the cardio-protective benefits in DCM of these plant extracts might be partly achieved through their anti-ferroptotic effects, but these still need further investigation (**Table 3**).

**Table 3:** The effects of plant extracts on ferroptosis and DCM.

Plant extract	DCM protective evidences	Anti-ferroptosis in other model/tissue	Anti-ferroptosis in DCM
Resveratrol	Yes (116, 117)	Cardiac I/R models *in vivo* and *in vitro* (118, 119)	Not available
Flavonoids:	Yes (120)	LPS-stimulated myocardial injury (122); Cardiomyocyte ferroptosis model (123); Fatty liver disease (124)	Not available
Sulforaphane:	Yes (38, 128–131)	Diabetic liver (132); Cardiac arrest and resuscitation (133); Cardiac I/R models (127)	AMPK/Nrf2 activation (38)
Curcumin:	Yes (134–136)	Osteoarthritis (137); Acute kidney injury (138); Cigarette smoke caused lung epithelial injury (139)	Increased function of Nrf2, and expression of GPX4 and HO-1 (55)
Berberine:	Yes (140, 141) (142–144)	Islet beta cells loss in T1DM (145); PCOS cell model (146); Contrast-induced nephropathy (147); Bone loss induced by NAFLD (148)	Not available

I/R, ischemia/reperfusion; LPS, lipopolysaccharides; T1DM, type 1 diabetes mellitus; PCOS, polycystic ovarian syndrome; NAFLD, nonalcoholic fatty liver disease; AMPK, AMP-activated protein kinase; Nrf2, nuclear factor erythroid-2-related factor-2; HO-1, heme oxygenase-1.

## 7 Ferroptosis inhibitors and DCM

Ferrostatin-1 (Fer-1) and liproxstatin-1 (Lip-1) are ferroptosis inhibitors and achieve their effects by suppressing lipid peroxidation (149). Fer-1 ameliorates cardiac injury caused by lipopolysaccharide (150), H2O2 (150, 151), isoproterenol (152), 5-fluorouracil (153), and doxorubicin (154). Lip-1 has been found to reduce cardiomyocyte ferroptosis induced by heat shock (155), 2,3,7,8-Tetrachlorodibenzo-p-dioxin (156), and I/R injury (138). Furthermore, Fer-1 was found to inhibit cardiomyocyte ferroptosis induced by palmitic acid (40, 102). Both Fer-1 (40) and Lip-1 (38) were found to be effective in inhibiting ferroptosis in DCM (**Figure 1**).

## 8 Conclusion and perspectives

As a severe complication of diabetes, the mechanisms underlying DCM’s pathogenesis and relative therapy strategies have drawn attention in recent years. Due to the unique high energy and high iron demand of heart tissue, both energy and iron dyshomeostasis have been found in DCM. Ferroptosis is novel cell death induced by iron overload and iron-dependent lipid peroxidation. In this review, we summarized the evidence on iron metabolism and ferroptosis in DCM, in particular the role of mitochondria in iron metabolism. However, detailed and in-depth evidence of the contribution of ferroptosis in DCM is still lacking. Insulin resistance plays key role in the pathogenesis of DCM; however, thus far, whether insulin resistance could trigger ferroptosis has never been explored in DCM. The causal relationship of mitochondria, the most important organelle for energy metabolism in cardiomyocytes, with iron metabolism and ferroptosis in DCM has been scarcely investigated. *In vivo* data on the effects of ferroptosis inhibitors on cardiac function is lacking, although they have been found to be protective in DCM models. In addition, some anti-diabetic drugs that are potentially cardio-protective in DCM might possess anti-ferroptotic effects, but this still needs to be confirmed by more direct research, both *in vivo* and *in vitro*. Clinical evidence for ferroptosis-related screening and therapy in DCM patients is also lacking. Therefore, the existing evidence on the role of ferroptosis in DCM is the tip of the iceberg, as more studies on the detailed mechanisms underlying the role of ferroptosis and regulation pathways in DCM are warranted. Targeting ferroptosis might provide more perspectives for DCM therapy but this still needs to be further explored.
